# Elements Involved in the Rsv3-Mediated Extreme Resistance against an Avirulent Strain of Soybean Mosaic Virus

**DOI:** 10.3390/v10110581

**Published:** 2018-10-24

**Authors:** Mazen Alazem, Kuan-Chieh Tseng, Wen-Chi Chang, Jang-Kyun Seo, Kook-Hyung Kim

**Affiliations:** 1 Department of Agricultural Biotechnology, College of Agriculture and Life Sciences, Seoul National University, Seoul 08826, Korea; m.alazem@gmail.com; 2 Plant Genomics and Breeding Institute, College of Agriculture and Life Sciences, Seoul National University, Seoul 08826, Korea; 3 Department of Life Sciences, National Cheng Kung University, Tainan 701, Taiwan; kevin9953@yahoo.com.tw (K.-C.T.); sarah321@mail.ncku.edu.tw (W.-C.C.); 4 College of Biosciences and Biotechnology, Institute of Tropical Plant Sciences, National Cheng Kung University, Tainan 701, Taiwan; 5 Department of International Agricultural Technology and Institutes of Green Bio Science and Technology, Seoul National University, Pyeongchang 25354, Korea; jangseo@snu.ac.kr; 6 Research Institute of Agriculture and Life Sciences, College of Agriculture and Life Sciences, Seoul National University, Seoul 08826, Korea

**Keywords:** extreme resistance, *Rsv3*, soybean mosaic virus, signal transduction pathways

## Abstract

Extreme resistance (ER) is a type of *R*-gene-mediated resistance that rapidly induces a symptomless resistance phenotype, which is different from the phenotypical *R*-resistance manifested by the programmed cell death, accumulation of reactive oxygen species, and hypersensitive response. The *Rsv3* gene in soybean cultivar L29 is responsible for ER against the avirulent strain G5H of soybean mosaic virus (SMV), but is ineffective against the virulent strain G7H. Rsv3-mediated ER is achieved through the rapid accumulation of callose, which arrests SMV-G5H at the point of infection. Callose accumulation, however, may not be the lone mechanism of this ER. Analyses of RNA-seq data obtained from infected soybean plants revealed a rapid induction of the abscisic acid pathway at 8 h post infection (hpi) in response to G5H but not to G7H, which resulted in the down-regulation of transcripts encoding β-1,3 glucanases that degrade callose in G5H-infected but not G7H-infected plants. In addition, parts of the autophagy and the small interfering (si) RNA pathways were temporally up-regulated at 24 hpi in response to G5H but not in response to G7H. The jasmonic acid (JA) pathway and many WRKY factors were clearly up-regulated only in G7H-infected plants. These results suggest that ER against SMV-G5H is achieved through the quick and temporary induction of ABA, autophagy, and the siRNA pathways, which rapidly eliminate G5H. The results also suggest that suppression of the JA pathway in the case of G5H is important for the Rsv3-mediated ER.

## 1. Introduction

Plants are equipped with several antiviral defense arrays that provide varying levels of resistance/tolerance depending on the specificity of the infecting virus [1,2,3,4]. One of the most characterized resistance mechanisms is the dominant *R*-gene resistance, whose product can directly or indirectly recognize specific viral effectors (usually viral proteins) [4,5]. 

There are two types of *R*-genes. The products of one type inhibit virus infection by targeting one or more stages in the infection cycle. One example of this type is the protein encoded by the tomato gene *TM-1*, which binds to the replicase of tomato mosaic virus (ToMV) and inhibits its replication [6,7]. The other type of *R*-gene acts earlier than the first by initiating effector-triggered immunity (ETI) upon the recognition of viral effectors. ETI leads to the suppression of viral replication/spread at the site of infection by inducing programmed cell death (PCD), which is manifested by necrotic lesions that indicate a hypersensitive response (HR). This second type of resistance is positively regulated by the stress hormone salicylic acid (SA) and may also involve the antiviral small interfering (si) RNA pathway [2,8,9,10,11].

Extreme resistance (ER) is a unique type of dominant resistance that results in early and rapid arrest of virus replication and spread, but seldom generates defensive symptoms such as PCD, HR, or accumulation of reactive oxygen species (ROS) [1,5,12]. ER has been reported for several potyviruses infecting different plants [13,14], for potato virus X (PVX) in Rx-resistant plants [15,16], and for the Y-strain of cucumber mosaic virus (CMV) in RCY1-transegenic *Arabidopsis* [17]. With respect to the current study, ER has also been reported against the G5H avirulent strain of soybean mosaic virus (SMV) in the soybean cultivar L29 [12,18]. ER against G5H is triggered when the Rsv3 protein, encoded by the *R*-gene *Rsv3* in cultivar L29, indirectly recognizes the cylindrical inclusions (CI) of the virus. The triggered ER is partially regulated by the *PP2C3a* gene, which controls the rapid accumulation of callose at the points of infection, leading to a swift arrest of GH5 spread [12]. Although the virulent strain G7H shares high homology with G5H, the substitution of a few amino acids in the CI region compromises the Rsv3-mediated ER [12,19]. This ER against SMV is unusual because *PP2C3a* is involved in the abscisic acid (ABA) signaling pathway. However, other reported examples of ER involve the SA pathway, such as the ER against CMV-Y in *A. thaliana,* or the ER against tomato bushy stunt virus (TBSV) in TM-2^2^-trasgenic tobacco [12,20]. The ER against CMV-Y and TBSV is negated if the SA production or signaling pathway is impaired.

Although callose accumulation arrests virus spread at the site of infection and is thus important in the ER against SMV-G5H, this mechanism may not explain the entire resistance response, because PCR failed to detect G5H in healthy-looking “G5H-infected” leaves [19]. The latter finding suggests that other cellular mechanisms might contribute to the elimination of G5H-related RNAs and proteins from infected cells.

As noted in the previous paragraphs, ER has been documented against several viruses in different hosts. A detailed understanding of how ER is regulated, however, is lacking. More specifically, there is little information on the downstream signaling involved in ER. A better understanding of how plants deploy ER will require studies that determine the changes at the transcriptome level at an early stage of infection. In the current study, we used RNA-seq data to analyze defense responses regulated by ER in soybean cultivar L29, which expresses ER against SMV-G5H through the *Rsv3* resistance gene. We also compared the responses of L29 to SMV-G5H, which fails to infect L29, and SMV-G7H, which rapidly infects L29. This comparison enabled us to identify genes/networks in L29 that are specifically regulated in response to SMV-G5H. 

## 2. Materials and Methods

### 2.1. Soybean RNA-Seq Data

RNA-seq data from soybean resistance cultivar L29 (carrying *Rsv3*) were obtained previously [12]. In brief, libraries were generated from untreated healthy plants, mock-treated plants, SMV-G5H-infected plants, and SMV-G7H-infected plants. The mock-treated and SMV-infected plants were sampled at 8, 24, and 54 h post infection (hpi), resulting in the generation of 10 libraries.

### 2.2. Annotation of Gene Functions

Soybean (*Glycine max*) annotation v1.0 on assembly v2.0 was used from the Phytozome v.12.1.5 release of December 2017 https://phytozome.jgi.doe.gov/pz/portal.html# and from EXPath databases [21]. Information on the RNA-seq data, the genes involved, and their annotated functions are provided in Table S4.

### 2.3. Pathway Analyses

RNA-seq data were subjected to bioinformatics analyses using EXPath tool in order to obtain lists of deferentially expressed genes (DEGs) (fold-change ≥2 or ≤0.5 and *p* ≤ 0.01) [21]. DEGs unique to G5H-inoculated plants (i.e., DEGs that were not shared by mock-inoculated or G7H-inoculated plants) were analyzed for their Pathway Enrichment profiles (Table S3). All pathways with *p* > 0.05 were excluded. 

### 2.4. Venn Diagrams and Heatmaps

Venn diagrams of DEGs were generated using the online tool (http://bioinformatics.psb.ugent.be/webtools/Venn/). Heatmaps for genes involved in hormone pathways were generated by Heatmapper [22].

### 2.5. ABA Treatment

The first trifoliate leaves of L29 line (age ~ 16 days) were sprayed with ABA (100 µM) or Mock (0.1% MeOH) 24 h prior to SMV-G7H-GFP inoculation, plants received another ABA treatment two days post infection (dpi), and samples were collected 5 dpi for analysis.

### 2.6. Virus Infection 

The two unifoliate leaves from L29 plants were infected with 10 µg/leaf pSMV-G7H-GFP plasmid [23]. A pool of Systemic leaves from 4 plants were mixed and divided into 0.1 g aliquots as source of virus inocula. One mL of phosphate buffer was vortexed with the 0.1 g infected tissues and centrifuged for 10 min at 13,000 rpm. 50 µL from the supernatant was rub-inoculated onto each leave of the trifoliate, and samples were collected 5 dpi for analysis.

### 2.7. RNA Analysis

Total RNA was extracted using RNA-extraction kit (Bio Cube, Suown, South Korea) following manufacturer’s instruction. One µg of total RNA was used for cDNA synthesis using GoScript kit (Promega, Madison, WI, USA). Real-time quantitative RTq-PCR was carried out with SYBR-Green (Promega) to measure the relative expression of target genes using ΔΔCT method. Actin11 was used as internal control, and primer used in this study were listed in Table S5.

### 2.8. Protein Blot

Total protein was extracted from 0.1 g of inoculated leaf pool from three plants as described previously [23]. Detection of SMV-G7H-GFP was carried out with protein blot using poly-colonal anti-GFP antibody (Sigma, St. Louis, MO, USA), and ponceau-S was used as loading control.

## 3. Results

### 3.1. Deferentially Expressed Genes Indicate Rapid Induction of the ABA Pathway

The RNAseq data was first validated by RTqPCR using eight different genes (Table S1). Expression levels of all tested genes were close to their levels obtained from the RNAseq data, therefore confirming the reliability of the RNAseq results.

**Figure 1:**
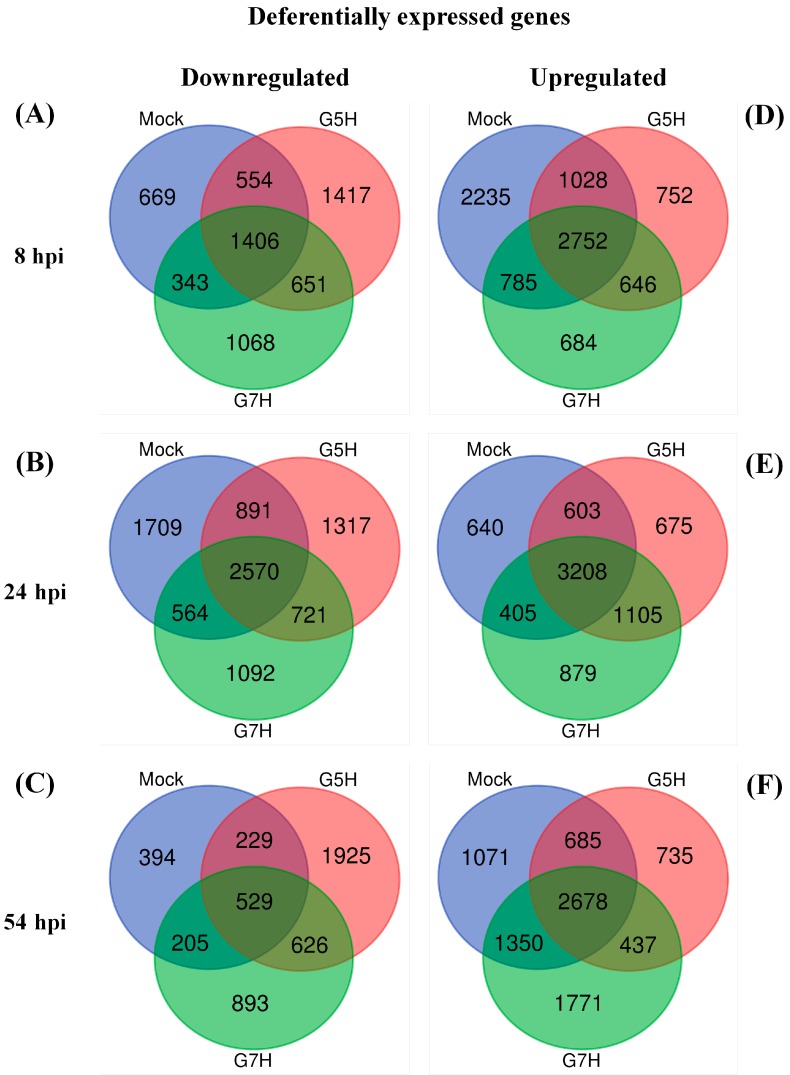
Venn diagrams illustrating the clustering of the differentially expressed genes (DEGs). The diagrams show up- and downregulated DEGs from soybean plants (cultivar L29 carrying the *Rsv3* ER gene) that were mock inoculated, infected with SMV avirulent G5H, or infected with SMV virulent G7H. DEGs were subjected to calculation of intersection using Venn diagrams: Downregulated DEGs at 8 hpi (**A**), 24 hpi (**B**), and 54 hpi (**C**). Upregulated DEGs at 8 hpi (**D**), 24 hpi (**E**), and 54 hpi (**F**). DEG lists were obtained from RNA-seq data using EXPath Tool by comparing the expression levels of healthy plants to those in mock plants and G5H- and G7H-infected plants at different time points.

As noted earlier, soybean cultivar L29 exhibits ER against the SMV avirulent strain G5H, but is highly susceptible to the virulent strain G7H. To determine which genes or defense mechanism/pathways are regulated upon infection with the avirulent strain G5H, we generated lists of DEGs by comparing expression in mock-treated, G5H-treated, and G7H-treated plants with the expression in healthy plants using EXPath Tool, a web-based platform that integrates gene expression data with metabolic pathways [21]. DEGs of mock-, G5H-, and G7H- treated plants at 8, 24, and 54 hpi were subjected to calculation of intersection. Venn diagrams showed that down-regulated DEGs were more abundant in G5H- or G7H-treated plants than in mock-treated plants at all time points except for G7H at 54 hpi (Figure 1). This analysis was used to exclude genes that are commonly regulated following G5H, G7H, and mock treatment, and to identify DEGs unique to G5H-infected plants. 

**Figure 2:**
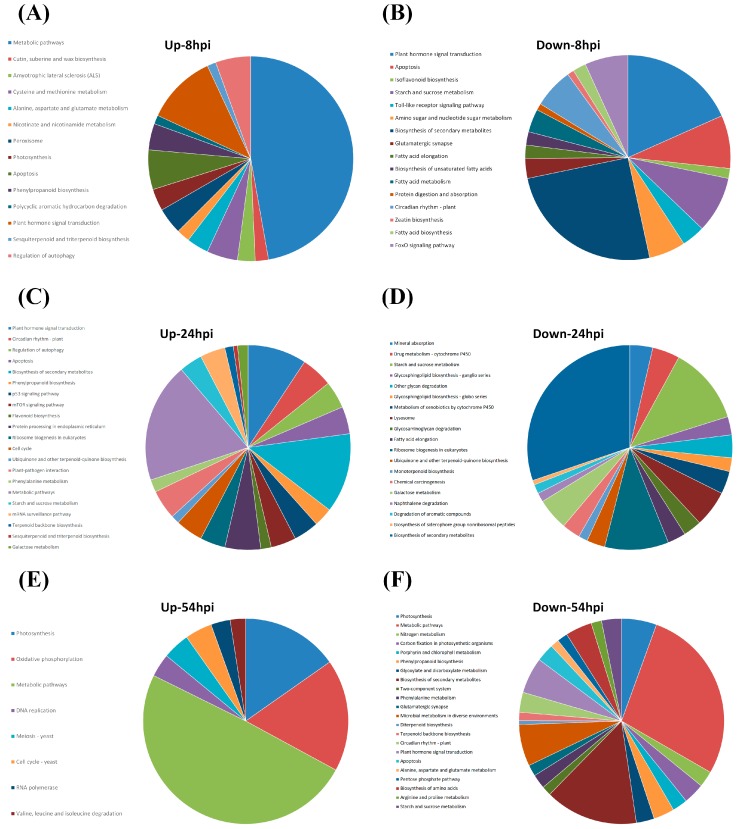
Relative distribution of enriched pathways in soybean plants (L29 *Rsv3*-cultivar) infected with SMV-G5H at different time points. Enriched pathways in the upregulated DEGs in G5H-infected plants at 8 hpi (**A**), 24 hpi (**C**), and 54 hpi (**E**). Enriched pathway in the downregulated DEGs in G5H-infected plants at 8 hpi (**B**), 24 hpi (**D**), and 54 hpi (**F**).

**Figure 3:**
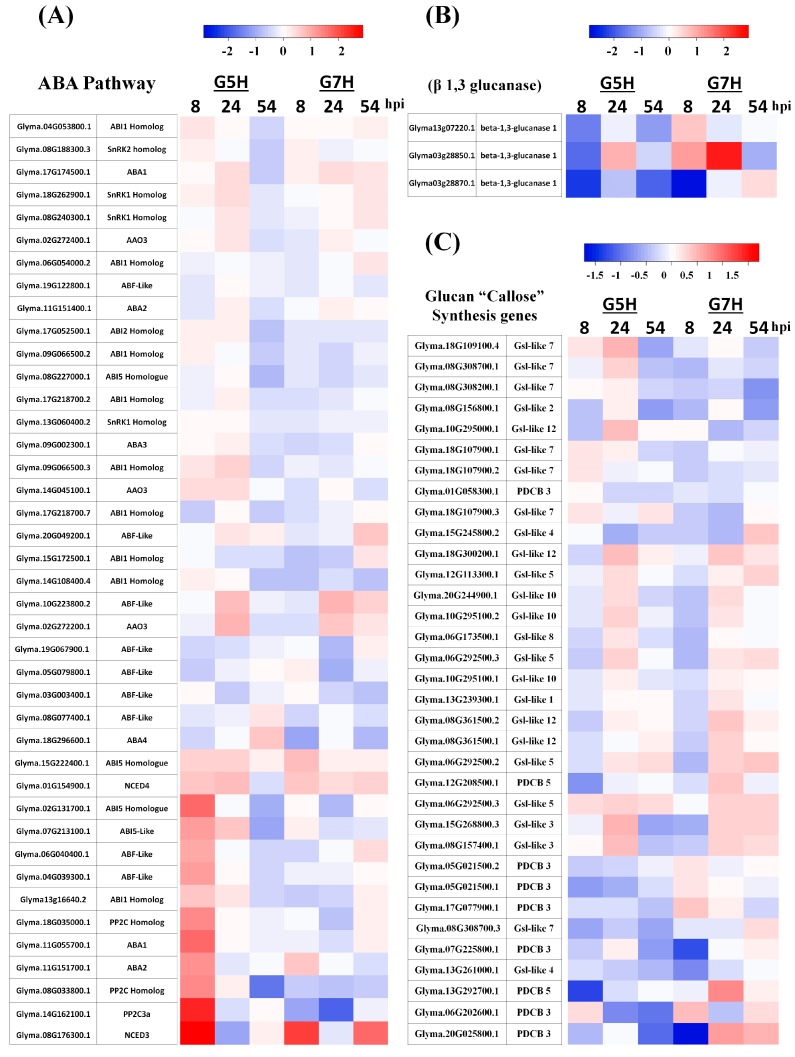
Heatmaps of genes involved in the ABA pathway, synthesis of β-1,3 glucanases and glucan in soybean plants (L29 *Rsv3*-cultivar) infected with SMV-G5H and SMV-G7H. Gene expressions from soybean plants infected with the avirulent SMV strain G5H and the virulent strain G7H at 8, 24, and 54 hpi were used to generate heatmaps for genes involved in the ABA pathway (**A**), genes that encode for β-1,3 glucanases (**B**), and glucan synthesis and plasmodesmata callose-binding proteins (*PCBPs*) (**C**). Red and blue indicate up- and down-regulation, respectively, in terms of the fold-change indicated by the scale above each heatmap.

The up-regulated DEGs unique to G5H infection (752, 675, and 735 genes at 8, 24, and 54 hpi, respectively; Figure 1D–F) and the down-regulated DEGs unique to G5H infection (1417, 1317, and 1925 genes at 8, 24, and 54 hpi, respectively; Figure 1A–C) were subjected to Pathway Enrichment Analyses in EXPath Tool [21]. Genes involved in metabolic pathways represented a large proportion of the DEGs at 8 and 54 hpi (Figure 2A,B,E,F). Interestingly, regulation of autophagy, apoptosis, and plant hormone signaling transduction were up-regulated at 8 and 24 hpi (Figure 2A,C). The top 15 up-regulated genes included several genes in the ABA pathway that were up-regulated at 8 hpi (Table S2). The finding of several up-regulated genes in the ABA-related pathway prompted us to investigate how the ABA pathway generally responded to infection by G5H and G7H. Several genes involved in ABA biosynthesis and signaling were induced early during G5H infection (Figure 3A). This rapid induction declined at 24 hpi, and the expression of several of these genes was reduced at 54 hpi. On the other hand, most of the ABA genes showed no-regulation or down-regulation following infection with G7H (Figure 3A). These data suggest that the ABA pathway is involved in the rapid ER response against G5H. Because ABA negatively regulates the transcription of β-1,3 glucanses [24,25], the enzymes that catabolize callose, we measured the expression levels of three genes that encode β-1,3 glucanses. The expression of *Glyma03g28850.1* was temporarily and slightly increased at 24 hpi, but the expression of the other two glucan genes was drastically reduced at 8 hpi in response to G5H infection, and remained relatively down-regulated at 24 and 54 hpi (Figure 3B). In response to G7H infection, *Glyma13g07220.1* and *Glyma03g28870.1* were differentially regulated at 8 hpi, but were unregulated at later times. The other transcript, *Glyma03g28850.1*, was up-regulated at 8 and 24 hpi, but its expression subsequently decreased (Figure 3B). 

Next, we determined whether SMV infection affects the expression of the glucan synthesis-like (*Gsl*) gene family, which is responsible for the synthesis of the callose deposited at different tissues and organelles [26,27]. We also determined whether SMV infection alters the expression of plasmodesmata callose-binding proteins (*PDCBs*) [28]. In response to G5H, many *Gsls* and *PDBPs* were down-regulated at 8 hpi (Figure 3C), but several orthologs of *Gsl*-like 3, 5, 7, 10, and 12 were up-regulated at 24 hpi. The majority of these genes were down- or unregulated at 54 hpi. These genes were also down-regulated in response to G7H at 8 hpi, but fewer *Gsl*-like genes where induced at 24 hpi by G7H. In addition, these genes were generally less-regulated in G7H than in G5H at 54 hpi (Figure 3C). 

With the exception of a few *Gsl*-*like-7* genes that were up-regulated in response to G5H at 24 hpi, the expression profiles of *Gsl*-like-7 genes were generally similar in response to G5H and G7H at 8 and 24 hpi (Figure 3C). The expression of several of these genes increased slightly at 54 hpi in response to G7H (Figure 3C). As noted earlier, callose deposition is a key factor in the ER against G5H [12]. The data obtained here suggest that the regulation of callose deposition might be post-translationally enhanced by the ABA-mediated down-regulation of β-1,3 glucanase.

**Figure 4:**
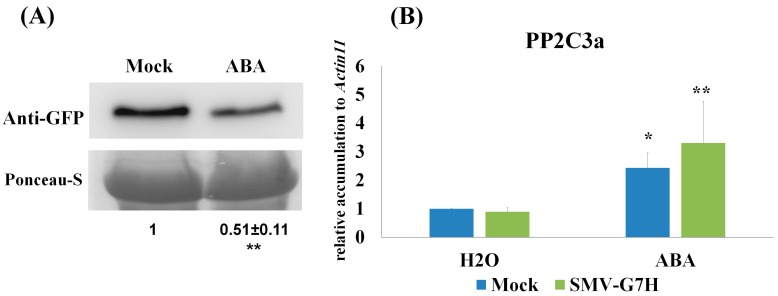
Effect of exogenous application of ABA on the SMV-G7H-GFP and *PP2C3a* levels in L29 soybean cultivar. (**A**) Protein blot for SMV-G7H-GFP in response to exogenous application of ABA (100 µM) or Mock (0.1% MeOH). Upper panel is GFP level, and lower panel is ponceau-S as loading control. (**B**) RTqPCR of *PP2C3a* gene in response to SMV-G7H-GFP infection, ABA, or a mix of both. *Actin11* was used as internal control. Data are means ± standard deviation from 3 biological replicates. Statistical analysis was carried out using one-sided student *t* test to determine the significance of the regulation compared to mock-treated plants (where * and ** indicates *p* < 0.05, and *p* < 0.01, respectively).

We previously reported that the *PP2C3a* gene is markedly upregulated in response to SMV-G5H infection, but not to SMV-G7H infection [12]. Overexpressing *PP2C3a* resulted in enhancing callose accumulation at plasmodesmata and arresting the spread both SMV strains G5H and G7H at the point of infection [12]. Since ABA was limitedly induced at early stage of G5H infection, but not in G7H, and that ABA regulates the expression of members of PP2C clade in other species such as *Arabidopsis thaliana* [29]. We hypothesize that exogenous application of ABA might induce resistance to the virulent strain G7H through *PP2C3a*. To test this, 100 µM of ABA was sprayed twice onto the first trifoliate leaves of L29; 24h before SMV-G7H infection, and 2 dpi. Indeed, ABA treatment decreased the accumulation of SMV-G7H by around 50% compared to control plants (Figure 4A). As shown previously [12], SMV-G7H did not induce *PP2C3a*, but ABA treatment increased the expression of *PP2C3a* by more than 3 folds (Figure 4B). These data showed that exogenous application of ABA can still induce partial resistance to the virulent strain through *PP2C3a*. However, since exogenous application of ABA did not completely block G7H accumulation, the results imply that in addition to ABA, other defense components may be required to accomplish the Rsv3-mediated ER.

### 3.2. Autophagy and the Antiviral siRNA Pathway Are Regulated in G5H-Infected Plants

**Figure 5:**
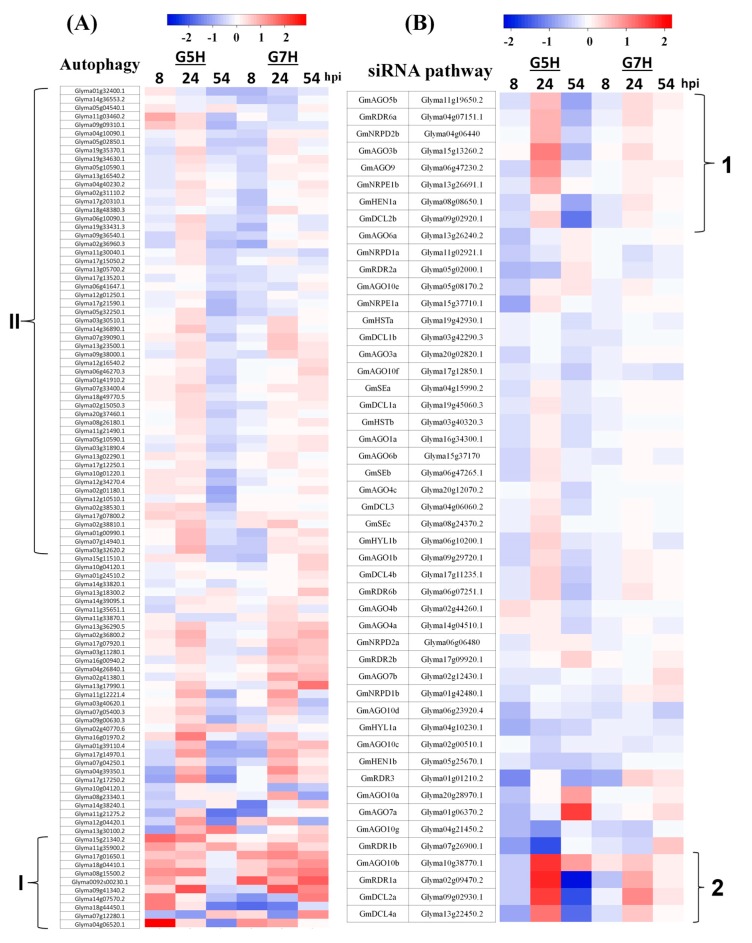
Heatmaps of genes involved in autophagy and siRNA pathways. Heatmaps for genes involved in authophagy (**A**) and the siRNA pathway (**B**) were generated as described in Figure 3 legend.

Antiviral defenses in plants include not only resistance gene-mediated responses, but also the antiviral RNA silencing and autophagy responses [30,31]. In order to analyze autophagy responses upon SMV infection, all genes involved in autophagy were extracted from the Phytozome database, and their expression levels were measured in G5H- and G7H-infected plants (Table S4). In G5H-infected plants, few genes involved in autophagy and apoptosis were regulated at 8 hpi (Figure 2A), but as many as 38 of these genes were up-regulated at 24 hpi (Figure 2C). Although the expression of most genes was unchanged at 8 hpi in G5H-infected plants, a small cluster (I) was differentially up-regulated in G5H-infected plants but not in G7H-infected plants at 8 hpi (Figure 5A). At 24 hpi, a large cluster of genes (II) became slightly up-regulated in G5H-infected plants, but their expression levels were almost unchanged in G7H-infected plants. The negative regulator of autophagy target of rapamycin (TOR) kinases began to be induced at this point (Table S3) [32,33]). While the majority of the autophagy genes became down-regulated at 54 hpi in G5H-infected plants, their expression increased in G7H-infected plants (Figure 5A). These data suggest that autophagy has a temporary role in soybean–SMV compatible and incompatible interactions.

Given reports linking ABA to the siRNA pathway [34,35], we examined whether the siRNA pathway is regulated in response to G5H and G7H infection. Genes in involved in this pathway were previously identified [36]. Most of the genes in the siRNA pathway were down-regulated in G5H-infected plants or unchanged in G7H-infected plants at 8 hpi. At 24 hpi, however, the expression of genes in two sectors significantly increased in G5H-infected plants (1 and 2), and the other genes were either not regulated or were slightly up-regulated (Figure 5B). The genes *AGO3b*, *AGO5b*, *AGO9*, *AGO10b*, *RDR1a*, *RDR6a*, *RDR2a*, *DCL2a*, *HEN1a*, *NRPD2b*, and *NRPE1b* were significantly up-regulated, and orthologs of many of these are important in the antiviral siRNA pathway [37,38]. The expression of genes in clusters 1 and 2 was less affected in G7H-infected plants than in G5H-infected, and the expression of the other genes was almost unchanged at all time points in G7H-infected plants.

With the evident regulation of autophagy and the antiviral siRNA pathway at 24 hpi in response to G5H, we determined whether the top 15 up-regulated genes at 24 hpi had potential roles in ER (Table S2). Orthologs of heat shock protein 21 (*Hsp21*), purple acid phosphatase 12 (*PAP12*), and glutamine-dependent asparagine synthase 1 (*ASN1*) were up-regulated by several fold at 24 hpi, and only *PAP12* remained up-regulated at 8 to 54 hpi in response to G5H, but not in response to G7H (Table S2). All of these genes have been reported to contribute to plant tolerance to biotic and abiotic stresses, and *PAP12* and *Hsp21* were reported to enhance tolerance to oxidative stress, which results from viral infection in several cases [39,40,41,42]. At 54 hpi, most of the genes that were up-regulated in response to G5H infection were photosynthesis-related (Table S2).

### 3.3. Other Defence Pathways May Have Opposite Effects on ER Against SMV

**Figure 6:**
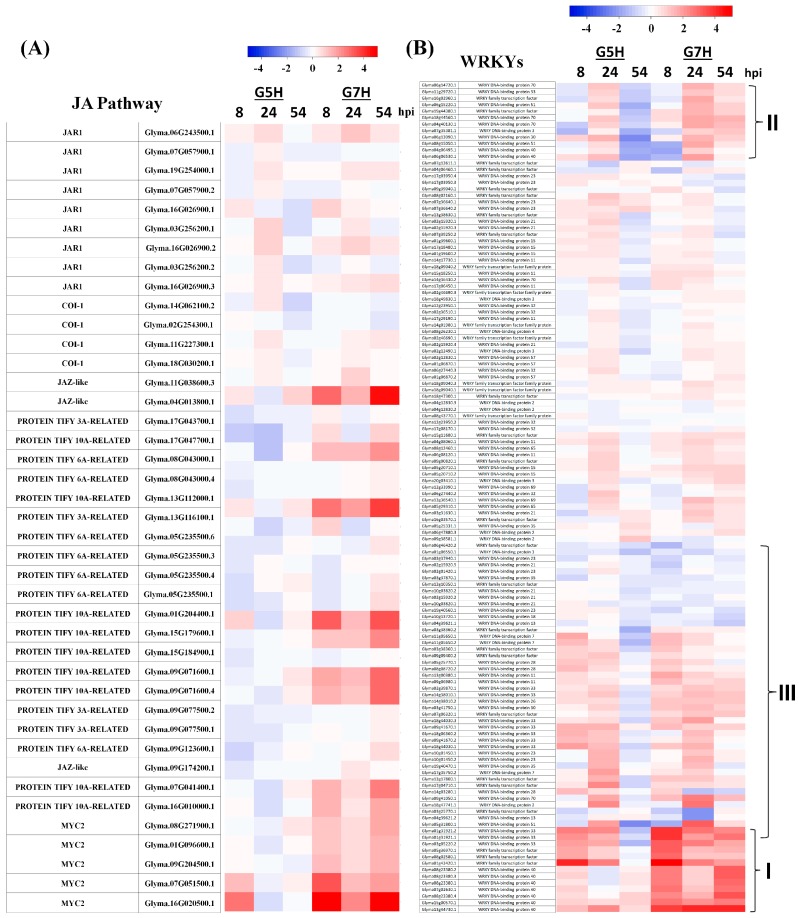
Heatmaps of genes involved in the JA pathway WRKY gene family. Heatmaps for genes involved in the JA pathway (**A**) and the WRKY gene family (**B**) were generated as described in Figure 3 legend.

Effects of the JA pathway on resistance to viruses has been controversial as they differ between compatible and incompatible interactions [2]. In the case of ER against G5H, most JA genes were un- or down-regulated, but they were up-regulated in response to G7H infection (Figure 6A), suggesting that suppression of JA pathway is required for ER resistance to G5H. The transcription factor gene family WRKY is also important in plant–pathogen interactions [43]. Expression of WRKY genes at 8 and 24 hpi did not significantly differ in response to G5H vs. G7H, except for a small cluster (I) that was strongly induced in response to G7H infection (Figure 6B). At 54 hpi, expression levels of several members of the WRKY family decreased in response to G5H (cluster II and III) but increased in response to G7H (Figure 6B). That these WRKY genes were up-regulated in response to G7H but down-regulated in response to G5H suggests that most of the WRKYs may negatively affect resistance against SMV strains.

### 3.4. SA, CKs, and BRs May Not Be Involved in ER Against G5H

Among the many reports that describe a central role for the SA pathway in resistance against viruses, a few describe ER [20,44]. Our RNA-seq data showed, however, that genes involved in the SA pathway have similar profiles in G5H-infected plants vs. G7H-infected plants (Figure S1). There was a mild up-regulation at 24 hpi for several SA pathway genes in response to infection by both strains, but at 54 hpi their expression had decreased in G5H-infected plants but not G7H-infected plants (Figure S1).

In compatible interactions, cytokinins (CKs) can increase plant tolerance to viruses by acting upstream of the SA pathway [2]. There was no clear induction of CK genes in response to G5H infection. Although cluster 1 showed a mild increase in G5H-infected plants at 8 hpi, it showed a similar increase in G7H-infected plants (Figure S3). Down-regulated clusters (2 and 3) were also similar between G5H-infected and G7H-infected plants at 24 and 54 hpi (Figure S3). These data suggest that CKs are not be involved in the ER against G5H.

Brassionsteroids (BRs) may also increase plant resistance to viruses in an SA-independent mechanism that is yet to be discovered [2]. In the current research, there was no clear clustering of BR genes that might be regulated in response to infection by G5H or G7H (Figure S3). Expression levels of most genes involved in the BR biosynthesis and signaling pathways showed mild to weak regulation in response to infection by either SMV strain. At 54 hpi, cluster I was downregulated in response to G5H but was unchanged in response to G7H, and cluster II was unchanged in response to G5H but was upregulated in response to G7H (Figure S3). These results indicate that CKs and BRs might not be involved in the ER-mediated resistance against SMV-G5H.

## 4. Discussion

ER allows plants to quickly terminate viral infections at the site of entrance. This instant reaction probably involves the induction of defense networks by transcriptional reprogramming. The published literature, however, includes little information on the ER-regulated mechanisms/pathways responsible for terminating virus replication at the cellular level. In the case of SMV-G5H, a clear aspect of the ER is the rapid induction of callose by *PP2C3a*, which stops G5H spread. [12,19]. Callose, however, does not scavenge SMV-G5H from infected cells, but infected tissues show no trace of SMV-G5H and look healthy [19]. In addition, induction of *PP2C3a* by ABA on L29 leaves was able to reduce G7H levels to ~50% (Figure 4A), yet, ABA was unable to eliminate G7H from infected tissue completely. This suggests that ER may regulate other defense mechanisms that help degrade and recycle SMV-related RNAs or proteins.

Townsend et al. [16] recently documented the very early events in the triggering of ER against PVX. The authors found that when Rx perceives the coat protein of PVX, it translocates to the nucleus where it binds with the golden-like (GLK) transcription factor. This interaction renders Rx specific in binding to DNA (specified by GLK-binding sites), thus allowing the transcriptional reprogramming that induces ER [16,45]. 

Our findings provide a general understanding of the second step, i.e., the transcriptional reprogramming, whereby an array of defense events are sequentially triggered within 24 h of infection, leading to the elimination of G5H. In addition, the susceptible case of G7H explained how the G7H-mediated transcriptional reprogramming affects specific pathways/networks that facilitate the rapid infection and spread of G7H. Moreover, when inducing the one of G5H-triggered defenses to G7H, plants exhibited a significant reduction in G7H levels.

We observed changes in metabolic pathways representing a large proportion of cellular processes during the first few hours of G5H infection (at 8 hpi, Figure 1A), which is probably a preparatory step for a larger following step. At this early stage, many genes involved in hormone signal transductions were also regulated (Figure 2A,B). Indeed, a rapid increase in transcripts encoding genes involved in the ABA pathway was observed (Figure 3A). In addition, transcriptional regulation in other hormone pathways was observed in responses to both G5H and G7H (Figure 5 and Figures S1–S3), which suggests that viral infection was behind the observed hormonal alterations in general, irrespective of the outcome of infection (susceptibility or resistance). Because the down-regulation of genes encoding β-1,3 glucanses is an expected consequence of the triggering of the ABA pathway (Figure 3B), this down-regulation probably explains the previously reported [12] rapid accumulation of callose at the site of G5H infection. Although reported cases of the positive roles of ABA in resistance to viruses mostly involve compatible interactions with dicots (the role of ABA in resistance is negative in rice) [2,34,35,46,47], the G5H-Rsv3 example represents a novel role for ABA in incompatible interactions and more specifically in ER. 

Callose accumulation may not be the only defense mechanism induced by ER, especially because callose is likely to provide only local restriction of SMV-G5H. Plants are also armed with other broad-spectrum defenses such as the antiviral siRNA pathway and autophagy. Each mechanism handles different substrates and can reduce virus levels depending on the amplitude of the mechanism [30,48]. 

Specific genes in the virus-derived (v)siRNA pathway were clearly induced in response to G5H infection at 24 hpi (Figure 5B). Orthologs of those genes in Arabidopsis (*RDR1a*, *RDR6a*, *DCL2a*, *DLC4a*, and a few *AGOs*) make up the backbone of the vsiRNA, and several reports have highlighted their antiviral functions [37,49,50,51,52]. This suggests that in ER, plants tend to limitedly induce the vsiRNA pathway once the viral replication intermediates (usually dsRNAs) are perceived. Therefore, vsiRNAs function in degrading viral genes only temporarily. This induction of vsiRNAs might also be linked to the earlier activation of ABA because ABA regulates the siRNAs in Arabidopsis resistance to bamboo mosaic virus [34,35]. 

Most of the genes involved in the ABA and siRNA pathways were down- or un-regulated in response to G7H infection (Figure 3A and Figure 4B), indicating that the virulent strain was able to block defense responses at a very early stage so that the infection could spread without obstacle. 

The JA pathway was reported to have positive roles in compatible interactions against few viruses such potato viruses X and Y [53,54], and no effects on others such as CMV-Y or oilseed rape mosaic virus [55]. However, its role in resistance in some incompatible interactions, such as that in *N*-tobacco resistant to TMV, is negative [56]. In the incompatible interaction with G5H, the JA pathway was clearly downregulated following infection, which suggests that its suppression is important for Rsv3-mediated ER. However, many genes in the JA pathway were markedly up-regulated in response to G7H at all sampling times (Figure 5A). Since JA enhances tolerance to several viruses in compatible interactions, this induction might be a way to increase tolerance to G7H as other defenses (siRNA and ABA) were unchanged or suppressed. Specific genes in the WRKY family might also help render plants susceptible to G7H, because clusters I and II were markedly up-regulated in G7H-infected plants but not in G5H-infected plants at 24 and 54 hpi (Figure 5B). However, how JA and WRKY regulate susceptibility or defense to SMV will require additional research.

Several autophagy and apoptosis genes were up-regulated in response to G5H infection at 8 hpi (Figure 2A and Figure 5A cluster I) and 24 hpi (Figure 5A cluster II). The early induction of autophagy may help the cell sweep viral particles into phagosomes before the particles become protected inside inclusion bodies [30,48]. In addition, the negative regulator of autophagy “TOR-like genes” was induced at 24 hpi (Figure 2A); the reduction of autophagy activity at that point might be a sign of decreasing stress. This was further evident from the general down-regulation of autophagy genes at 54 hpi (Figure 5A). Autophagy is normally suppressed by the negative regulation of TOR, but TOR is inhibited under stress, allowing autophagy to be activated [33]. Although cluster II of autophagy genes were up-regulated in response to G5H, their levels were down-regulated or weakly regulated in response to G7H (Figure 5A). The observation regarding autophagy genes is consistent with the hypothesis that the down-regulation of these genes in response to G7H may help explain the quick establishment of G7H infection. Thus, a temporary and limited activation of this system can help the infected cell recover itself from infection with less costs, since too much autophagy can may have opposite effects on resistance [30,48]. Further research is required to determine the degree to which autophagy helps soybean plants resist SMV.

In summary, Rsv3-mediated ER against SMV-G5H is evidently achieved in three steps: (1) the quick arresting of the virus at the site of infection by enhanced callose deposition; (2) the activation of the antiviral vsiRNA machinery to cleave SMV viral genes; and (3) the induction of autophagy to eliminate remaining viral components from the cell. 

In the susceptible interaction involving G7H, on the other hand, the virulent strain avoids RSV3-recognition (and thus downstream defenses) as a consequence of changes in a few amino acids in the CI region [19]. This allows G7H to re-design the cell to facilitate its rapid replication and spread. Obviously, induction of specific WRKYs may transcriptionally mediate the reduction or shutting down of defense networks (such as the ABA and the vsiRNA pathway). The induction of several autophagy genes in this case could be virus-derived to favor virus replication/movement as previously reported [48]. 

Our understanding of Rsv3-mediated ER in soybean L29 plants will be increased by the identification of factors that are associated with Rsv3 and that help trigger the ER response. 

## Supplementary Materials

The following are available online at http://www.mdpi.com/1999-4915/10/11/581/s1. Figure S1: Heatmap of genes involved in the SA pathway. Figure S2: Heatmap of genes involved in the cytokinin pathway. Figure S3: Heatmap of genes involved in the brassinosteroid pathway. Table S1: Validation of RNAseq data by RTqPCR. Table S2: Top G5H-upregulated genes in plants infected with SMV-G5H and SMV-G7H at 8, 24, and 54 hpi. Table S3: Enriched pathways of upregulated and downregulated DEGs in G5H-infected plants at 8, 24, and 54 hpi. Table S4: RNA-seq data of the following defence pathways examined in this study: ABA, SA, JA, Cks, BRs, siRNAs, WRKYs, Gsls, and β-1,3 glucanases. Table S5: Primers used in the study.
