# Supplementary figures and images for: Elements Involved in the Rsv3-Mediated Extreme Resistance against an Avirulent Strain of Soybean Mosaic Virus
Source: Viruses. 2018 Oct 24;10(11):581. doi: 10.3390/v10110581 (PMC6267276; doi:10.3390/v10110581)

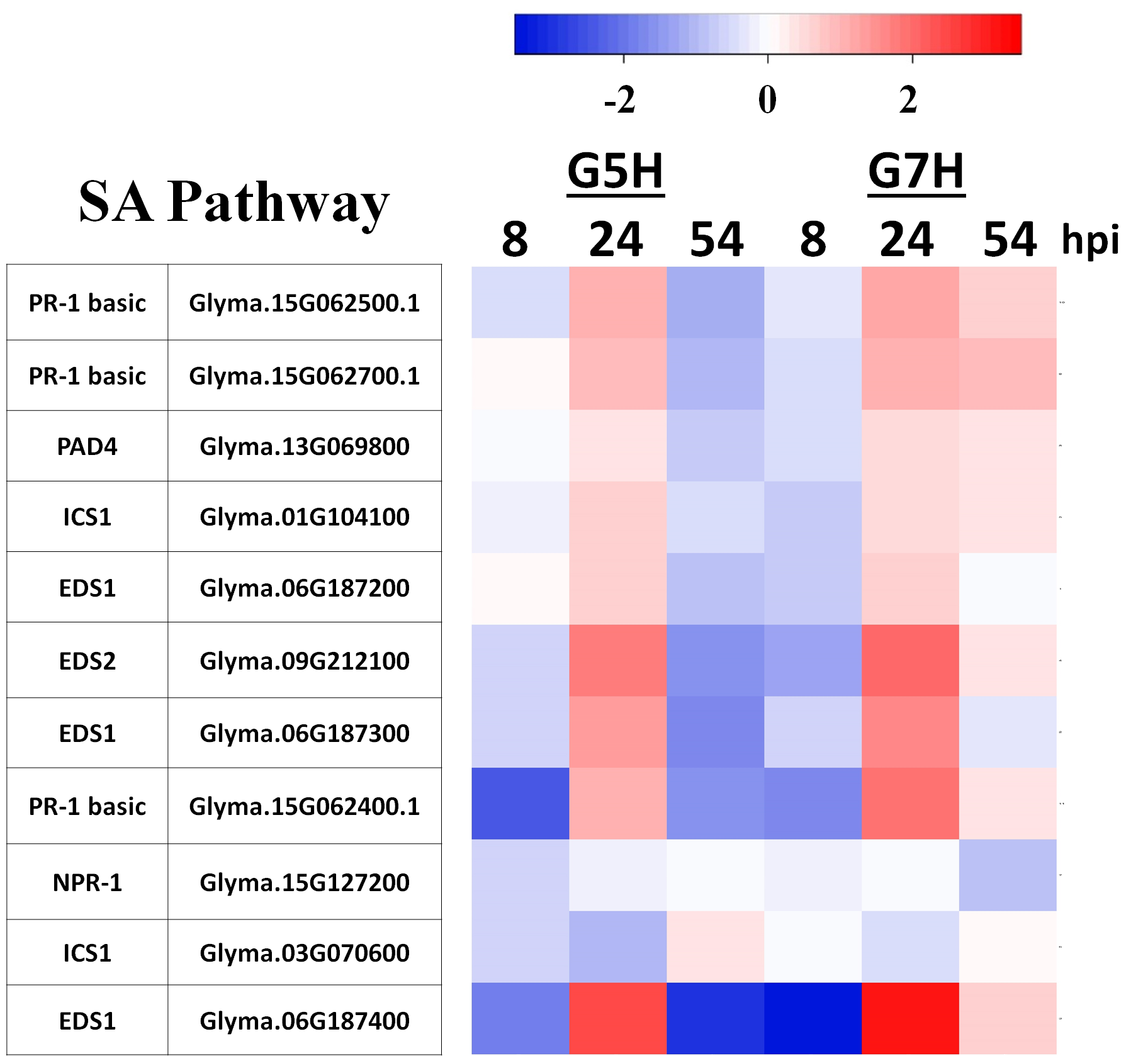

Supplement: Supplementary file 1 [file viruses-10-00581-s001.zip › 7. viruses-346980 suppl/S1.tif]

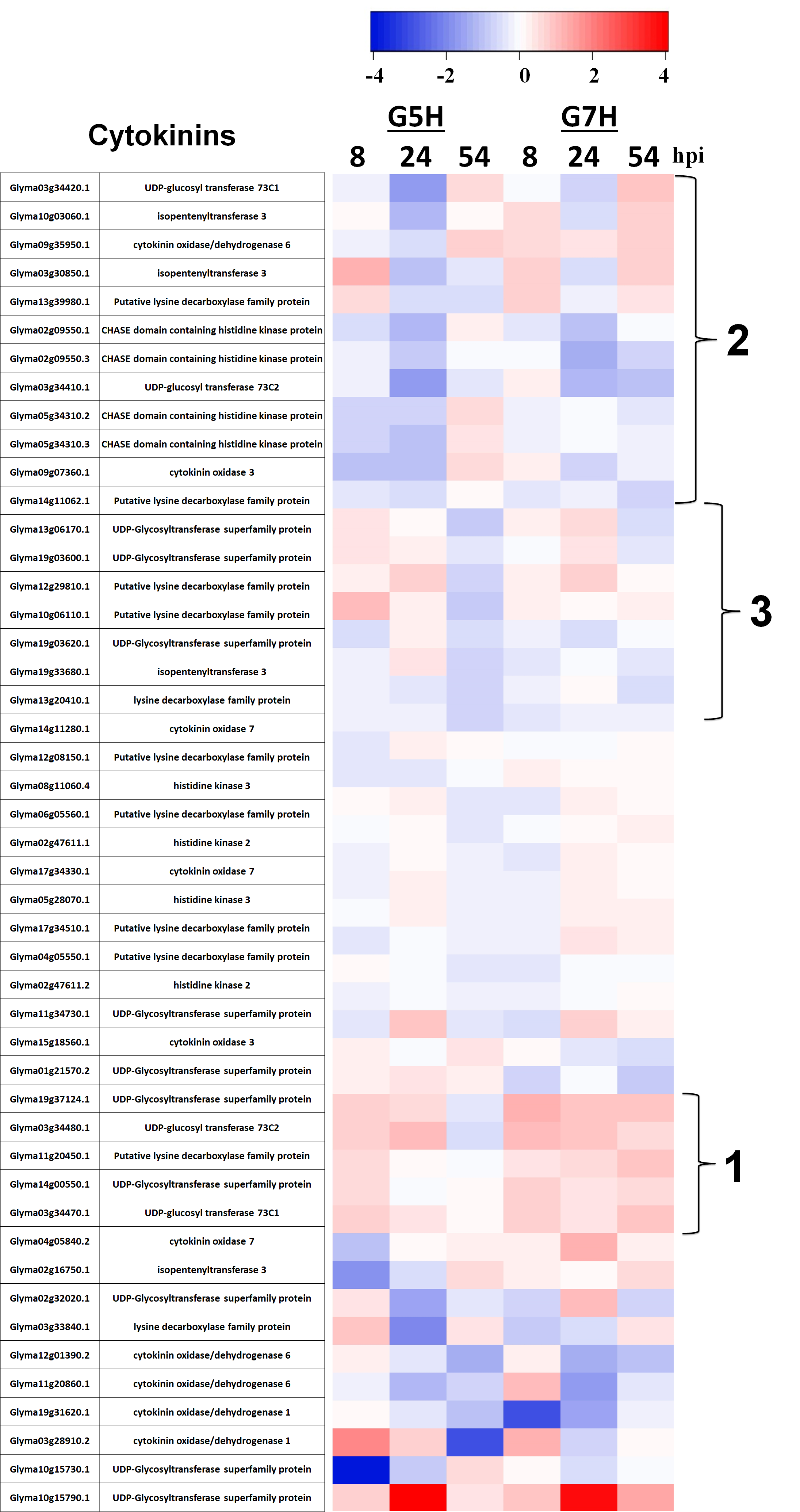

Supplement: Supplementary file 1 [file viruses-10-00581-s001.zip › 7. viruses-346980 suppl/S2.tif]

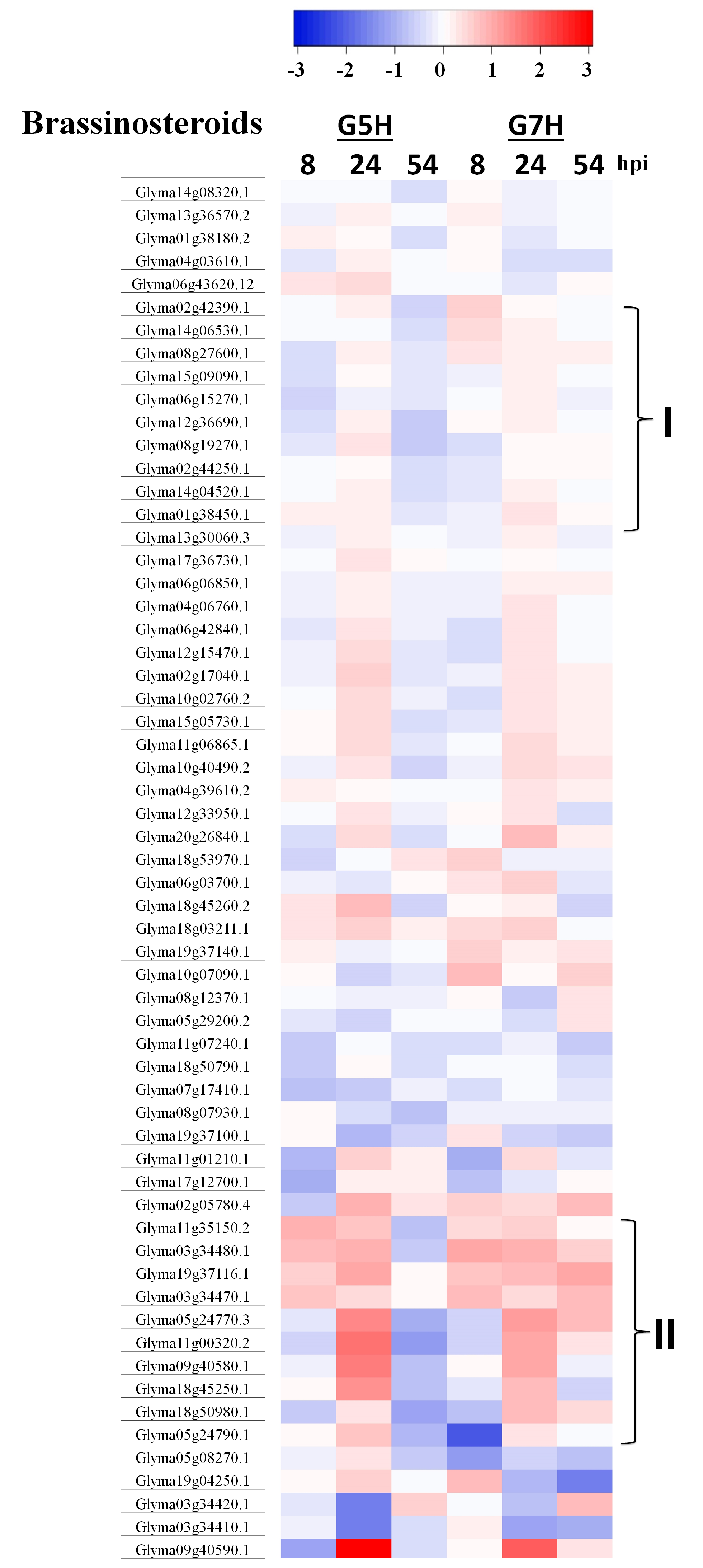

Supplement: Supplementary file 1 [file viruses-10-00581-s001.zip › 7. viruses-346980 suppl/S3.tif]
